# Successful treatment for pseudoaneurysm following distal pancreatectomy with celiac axis resection without postoperative pancreatic fistula: a case report

**DOI:** 10.1186/s40792-024-01914-w

**Published:** 2024-05-08

**Authors:** Kaito Fukuda, Ken Koyama, Yusuke Kyoden

**Affiliations:** Department of Surgery, Ibaraki Prefectural Central Hospital and Cancer Center, 6528 Koibuchi, Kasama, Ibaraki 309-1793 Japan

**Keywords:** Pancreatic cancer, Distal pancreatectomy with celiac axis resection, Pseudoaneurysm

## Abstract

**Background:**

Distal pancreatectomy with celiac axis resection (DP–CAR) represents an innovative surgical approach for locally advanced pancreatic body cancer in cases involving celiac axis invasion. However, this procedure carries significant perioperative risks, including arterial aneurysms and organ ischemia. Understanding these risks is crucial for optimizing patient outcomes and guiding treatment decisions.

**Case presentation:**

This case report describes a unique case of a 74-year-old male patient who was diagnosed with locally advanced pancreatic body cancer with invasion of the celiac and splenic arteries. He underwent DP–CAR after six cycles of chemotherapy. His postoperative course was uneventful without any evidence of postoperative pancreatic fistula. However, at the 10-month postoperative follow-up, pseudoaneurysm was incidentally detected in the anterior superior pancreaticoduodenal artery by follow-up computed tomography. It was successfully treated with coil embolization. He had no signs of tumor recurrence or relapse of pseudoaneurysm formation 2 years postoperatively. This case report discusses the potential risks of pseudoaneurysm formation in patients undergoing DP–CAR due to hemodynamic changes. We emphasize the significance of close monitoring in such cases.

**Conclusions:**

The case highlights the importance of recognizing and managing potential complications associated with DP–CAR in patients with pancreatic cancer. Despite its effectiveness in achieving complete resection, DP–CAR carries inherent risks, including the development of pseudoaneurysms. Vigilant surveillance and prompt intervention are crucial for optimizing patient outcomes and minimizing postoperative complications.

## Background

Pancreatic cancer remains a disease with high mortality rates and limited treatment options, particularly in cases of locally advanced disease. Managing such cases often requires a multidisciplinary approach that integrates surgical, medical, and interventional strategies. Distal pancreatectomy with celiac axis resection (DP–CAR) represents an innovative surgical procedure for locally advanced pancreatic body cancer, with a goal of achieving complete resection even in cases involving celiac axis invasion [1–3]. However, despite its potential benefits, DP–CAR is associated with perioperative risks, including alterations in vascular dynamics and the development of severe complications, such as arterial aneurysms and organ ischemia [2, 4–9]. Understanding the associated risks of DP–CAR is crucial for optimizing patient outcomes and guiding treatment decisions.

## Case presentation

A 74-year-old man with abdominal pain was referred to our hospital after a pancreatic mass was revealed on abdominal ultrasonography. He was subsequently diagnosed with pancreatic cancer with celiac and splenic artery involvement.

**Fig. 1:**
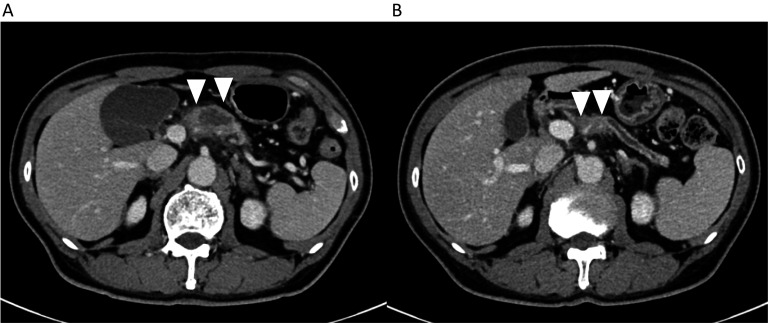
**A** Contrast-enhanced computed tomography shows the tumor before chemotherapy. **B** Contrast-enhanced computed tomography shows the tumor shrink from 3 to 2 cm after chemotherapy

He had no history of cardiovascular, arterial sclerotic diseases or diabetes. He was 168 cm tall, weighed 58 kg, and had a body mass index of 20.3 kg/m^2^. Blood test showed slight elevation in CA19-9 and DUPAN-2 levels. Abdominal contrast-enhanced computed tomography (CT) revealed a 3-cm pancreatic body tumor with main pancreatic duct dilation extending to the tail and significant parenchymal atrophy, particularly in the tail (Fig. 1A). The celiac artery was invaded 180°, with splenic vein occlusion. No lymph node metastasis was observed. Positron emission tomography CT did not reveal any distant metastases.

The patient was diagnosed with stage III pancreatic body cancer according to the Eighth edition of the AJCC Cancer Staging Manual [10]. The diagnosis was confirmed by ultrasound-guided endoscopic puncture aspiration. Six cycles of tegafur + gemcitabine hydrochloride (1000 mg/m^2^) were administered as neoadjuvant chemotherapy for borderline resectable pancreatic cancer. Post-chemotherapy blood tests showed a decrease in CA19-9 (53.2 → 7.8 U/mL) and DUPAN-2 (162 → 63 U/mL). Abdominal contrast-enhanced CT revealed tumor shrinkage from 3 to 2 cm in size, with no changes in the findings of arterial and venous invasion (Fig. 1B). We performed DP–CAR for stage III pancreatic body cancer.

**Fig. 2:**
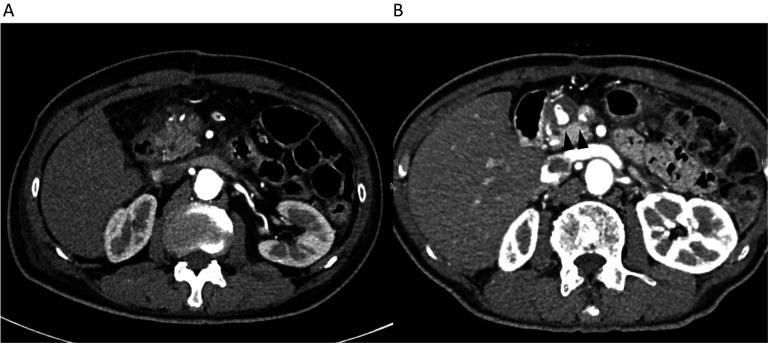
**A** Contrast-enhanced computed tomography revealed minimal fluid collection and no evidence of pseudoaneurysm on POD7. **B** Contrast-enhanced computed tomography revealing pseudoaneurysm in the anterior superior pancreaticoduodenal artery (ASPDA) (black arrowhead)

Surgery was successfully performed with an operation time of 371 min and intraoperative blood loss of 417 mL. We dissected the pancreas to the left of the portal vein using Endo GIA reinforced reload with Tri-Staple Technology®. Additionally, we dissected the celiac trunk after branching from the left gastric artery (LGA) and preserved the LGA. Contrast-enhanced CT on postoperative day 7 revealed minimal fluid collection (Fig. 2A). On the 16th postoperative day, the patient was discharged. Subsequently, he underwent regular check-ups, including blood tests and CT, with no significant findings up to 7 months postoperatively.

**Fig. 3:**
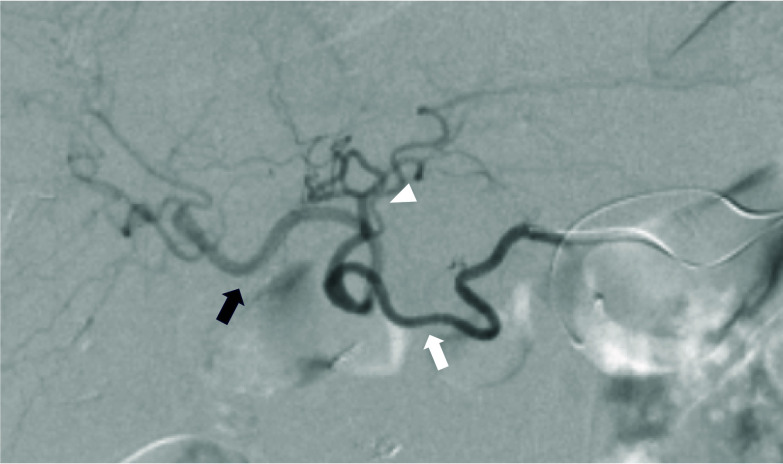
Left hepatic artery (white arrowhead) and right hepatic artery (black arrow) angiography via the left gastric artery (LGA) and right gastric artery (RGA) (white arrow)

**Fig. 4:**
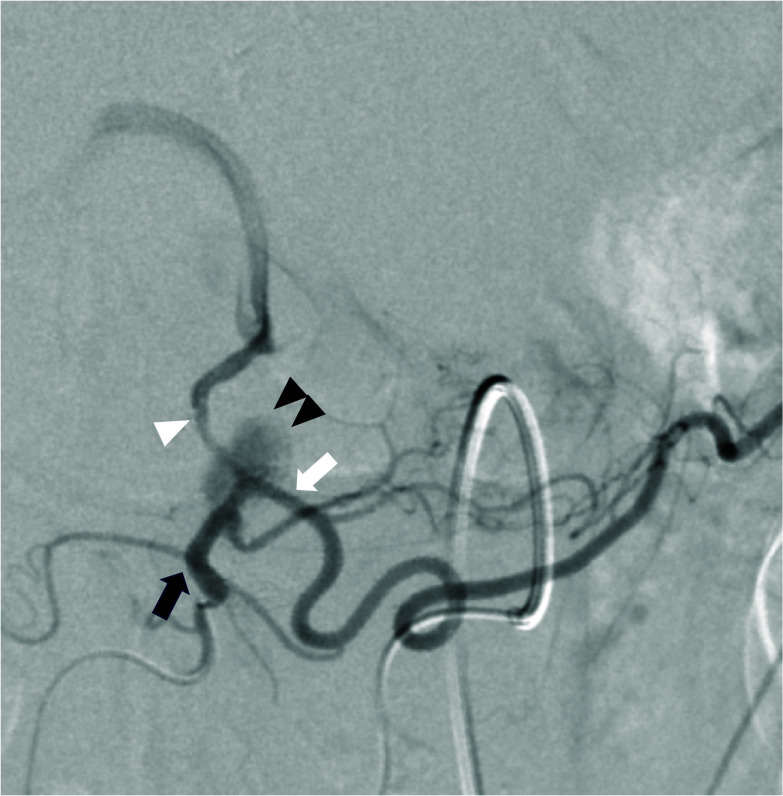
Superior mesenteric artery angiography via the ASPDA (black arrow). The bleeding point is detected (black arrowhead). An artery from the ASPDA just before the aneurysm is infra-pyloric artery (IPA). Blood flow of the GDA (white arrowhead) and right gastroepiploic artery (RGEA) (white arrow) is also confirmed

**Fig. 5:**
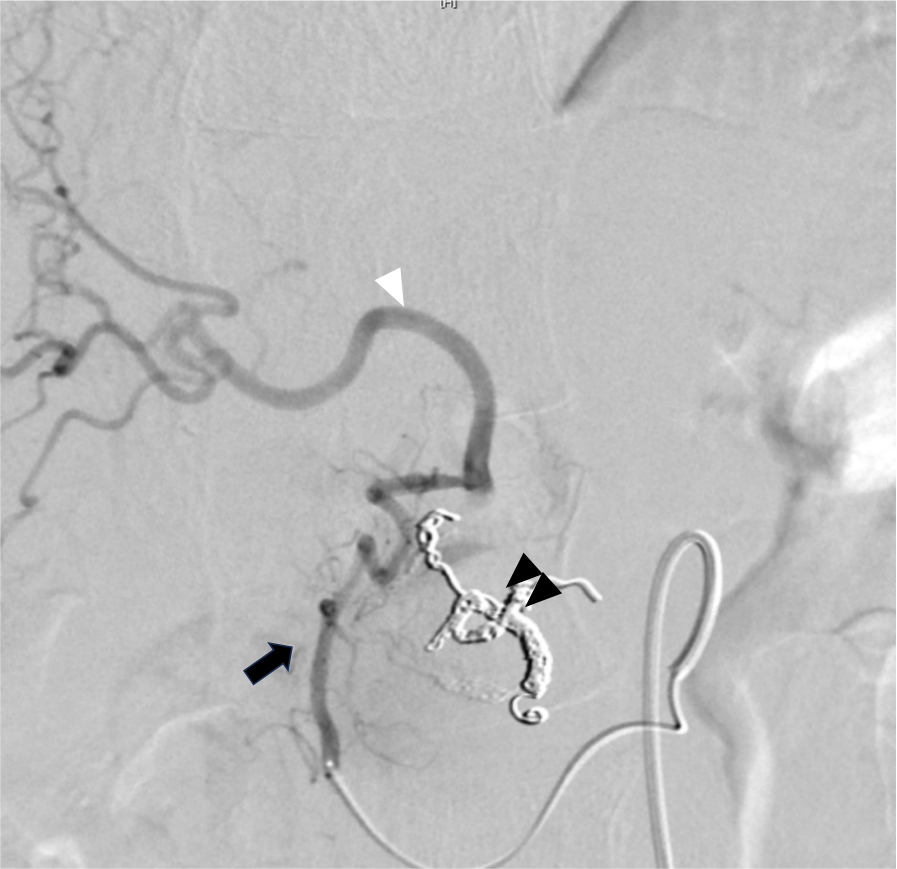
Coil embolization is performed for the ASPDA (black arrowhead), RGEA, IPA and GDA. Following embolization, posterior superior pancreaticoduodenal artery (black arrow) angiography is performed and shows blood flow in the right hepatic artery (white arrowhead)

**Fig. 6:**
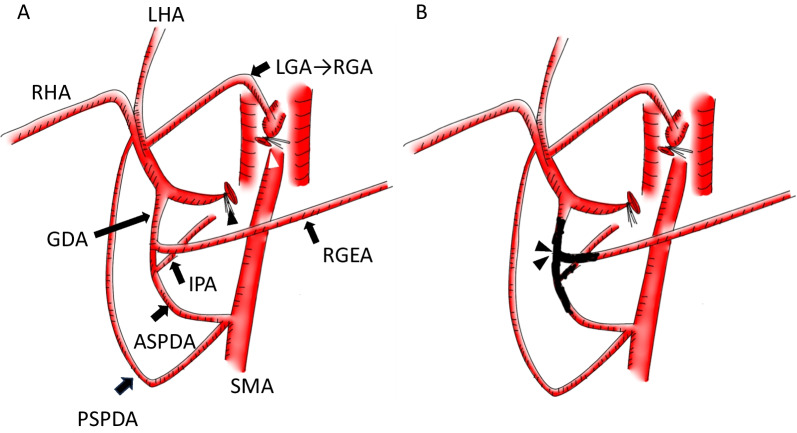
**A** Schema of arterial anatomy. Stump of CHA is indicated by black arrowhead and stump of celiac artery is indicated by white arrowhead. **B** Schema after the embolization. Coil embolization was performed for the ASPDA, GDA, IPA and GDA. The genuine location of the pseudoaneurysm was the junction of RGEA and ASPDA (blackout)

Postoperative follow-up CT at 10 months revealed a pseudoaneurysm that was incidentally detected in the anterior superior pancreaticoduodenal artery (ASPDA) (Fig. 2B), and an urgent angiogram was performed. First, hepatic artery angiography was performed through the left gastric artery and right gastric artery (RGA) (Fig. 3). Subsequently, to confirm the presence of the pseudoaneurysm in the ASPDA, angiography from the superior mesenteric artery (SMA) was performed (Fig. 4). Coil embolization was performed for the ASPDA, right gastroepiploic artery, infra-pyloric artery (IPA) and gastroduodenal artery (GDA) (Fig. 5). We also made the schema which describes the arterial anatomy, the location of pseudoaneurysm and embolization which we performed (Fig. 6). The patient had no tumor recurrence or relapse of pseudoaneurysm formation 2 years postoperatively.

## Discussion

DP–CAR is a surgical procedure for locally advanced pancreatic body cancer that enables complete resection even in cases of borderline resectable pancreatic body cancer with celiac axis invasion [1]. These patients demonstrate 1-, 2-, and 5-year overall survival rates of 81.1%, 56.9%, and 32.7% [2], respectively, with a median survival time ranging from 17.5 to 30.9 months [2, 3].

However, recognizing that this innovative surgical approach has the potential for severe complications owing to its alternations in vascular dynamics is imperative. The rate of Clavien–Dindo classification grade 3a or higher complications in DP–CAR is reported to be 41.3%, including gastric ischemia (28.8%), delayed gastric emptying (25%) [2], hepatic ischemia [4–6], bleeding [4, 5, 7], and the formation of arterial aneurysms [8, 9] related to pancreatic leakage.

In particular, intraperitoneal bleeding due to pancreatic leakage following DP–CAR is common and is a lethal complication [4, 5, 7–9]. In previous reports, there were two cases wherein the patients developed pseudoaneurysm rupture concomitant with pancreatic leakage requiring catheter intervention [8, 9]. However, to the best of our knowledge, this is the first report regarding pseudoaneurysm development without pancreatic leakage after a long period following DP–CAR. DP–CAR has a potential risk of pseudoaneurysm even without postoperative pancreatic fistula (POPF), and it may unexpectedly occur because of hemodynamic changes.

Theoretically, the hemodynamic changes induced by DP–CAR are similar to those induced by median arcuate ligament syndrome (MALS). This syndrome results in a relative increase in blood flow from the SMA and causes the development of arterial aneurysms in the pancreatic arcades. In particular, during pancreaticoduodenectomy, the presence of MALS requires special attention because ligating the GDA could disrupt collateral blood flow, potentially leading to severe complications such as hepatic or gastric ischemia [11, 12].

Although they have a similar hemodynamic background, differences exist regarding their treatment strategies. In general, MALS treatment involves dividing the median arcuate ligament until hepatic blood flow is confirmed. Arterial reconstruction is necessary if the hepatic blood flow is insufficient [13]. However, even in the absence of POPF or infectious fluid collection, arterial reconstruction following DP–CAR is not feasible. Therefore, in such cases, coil embolization seems to be the only treatment that preserves visceral blood flow, especially to the liver.

In this case, selective coil embolization was successfully performed without any complications for two reasons. First, the preservation of the LGA. As Okada et al. reported, preserving the LGA is important [14], and there are even reports which advocating for LGA reconstruction [15, 16]. The presence of blood flow from the LGA to both the stomach and liver is considered to be an important aspect. Second, we speculate that performing the surgery at high-volume centers where skilled interventional radiologists are available and capable of responding to any possible situations is desirable. The prior confirmation of blood flow from the RGA to the liver enabled successful GDA embolization. As emphasized in previous reports, microembolization that preserves arterial communications is indispensable [9]. This procedure requires delicate and highly skilled techniques.

Consequently, as pseudoaneurysm was incidentally detected and was preemptively and selectively treated, this patient was successfully rescued. Therefore, regular imaging follow-up is significant. DP–CAR is an excellent surgical procedure for locally advanced pancreatic body cancer; however, for a better prognosis, meticulous follow-up and skillful surgery are mandatory.

## Conclusion

We presented a case of pseudoaneurysm development following DP–CAR in a patient with pancreatic cancer without POPF. Considering the unique risks associated with this procedure, performing DP–CAR in specialized facilities and maintaining vigilant follow-up are essential.

## Data Availability

Not applicable.

## Abbreviations

DP–CAR: Distal pancreatectomy with celiac axis resection
CT: Computed tomography
ASPDA: Anterior superior pancreaticoduodenal artery
RGA: Right gastric artery
SMA: Superior mesenteric artery
GDA: Gastroduodenal artery
POPF: Postoperative pancreatic fistula
MALS: Median arcuate ligament syndrome
